# Supplementary material for: Seminoma and Embryonal Carcinoma Footprints Identified by Analysis of Integrated Genome-Wide Epigenetic and Expression Profiles of Germ Cell Cancer Cell Lines
Source: PLoS One. 2014 Jun 2;9(6):e98330. doi: 10.1371/journal.pone.0098330 (PMC4041891; doi:10.1371/journal.pone.0098330)
Supplement: File S1 — ZIP file containing DMRforPairs output for significant regions. Please start from the html files. (ZIP) [file pone.0098330.s008.zip › figures/29413.pdf]

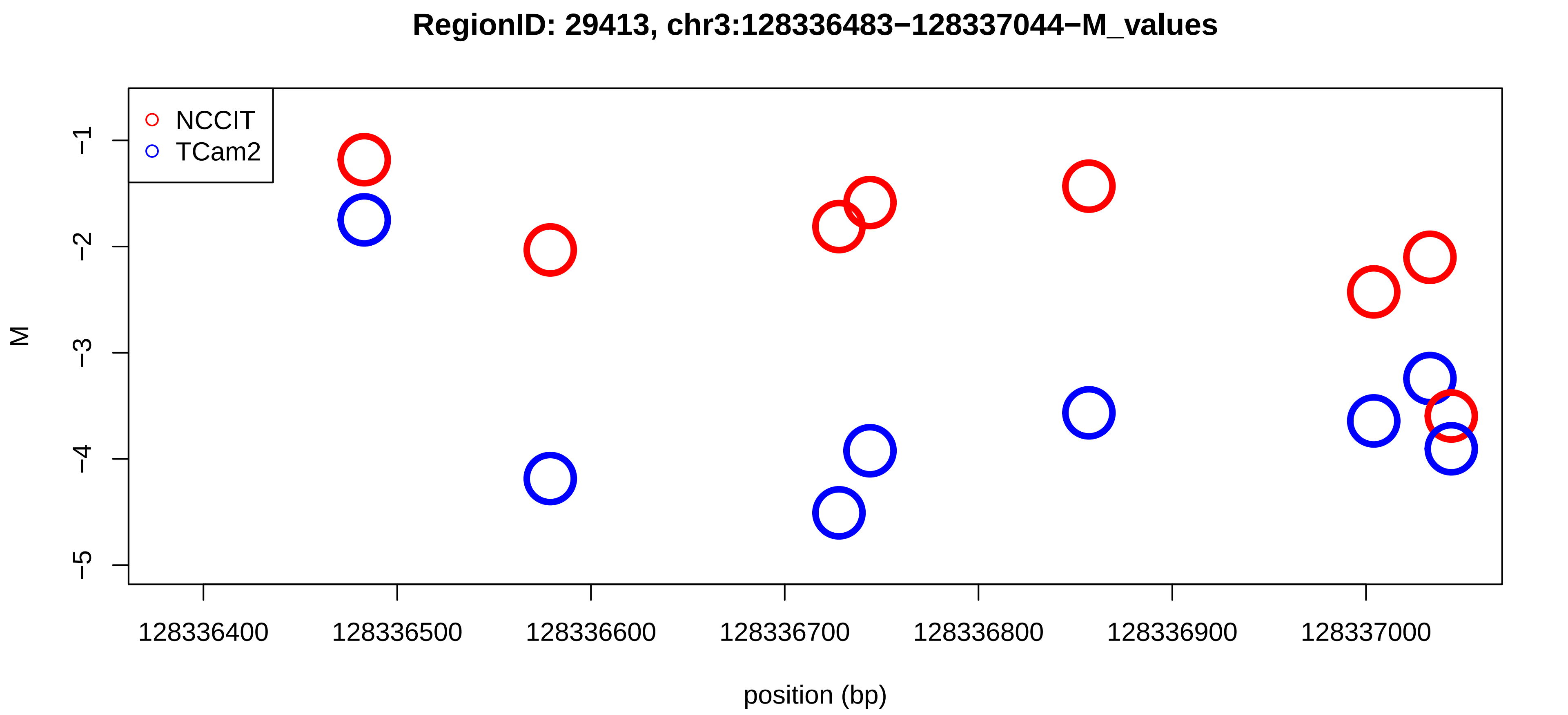

RegionID: 29413, chr3:128336483−128337044−M_values
NCCIT
TCam2
M
−1
−2
−3
−4
−5
128336400
128336500
128336600
128336700
128336800
128336900
128337000
position (bp)

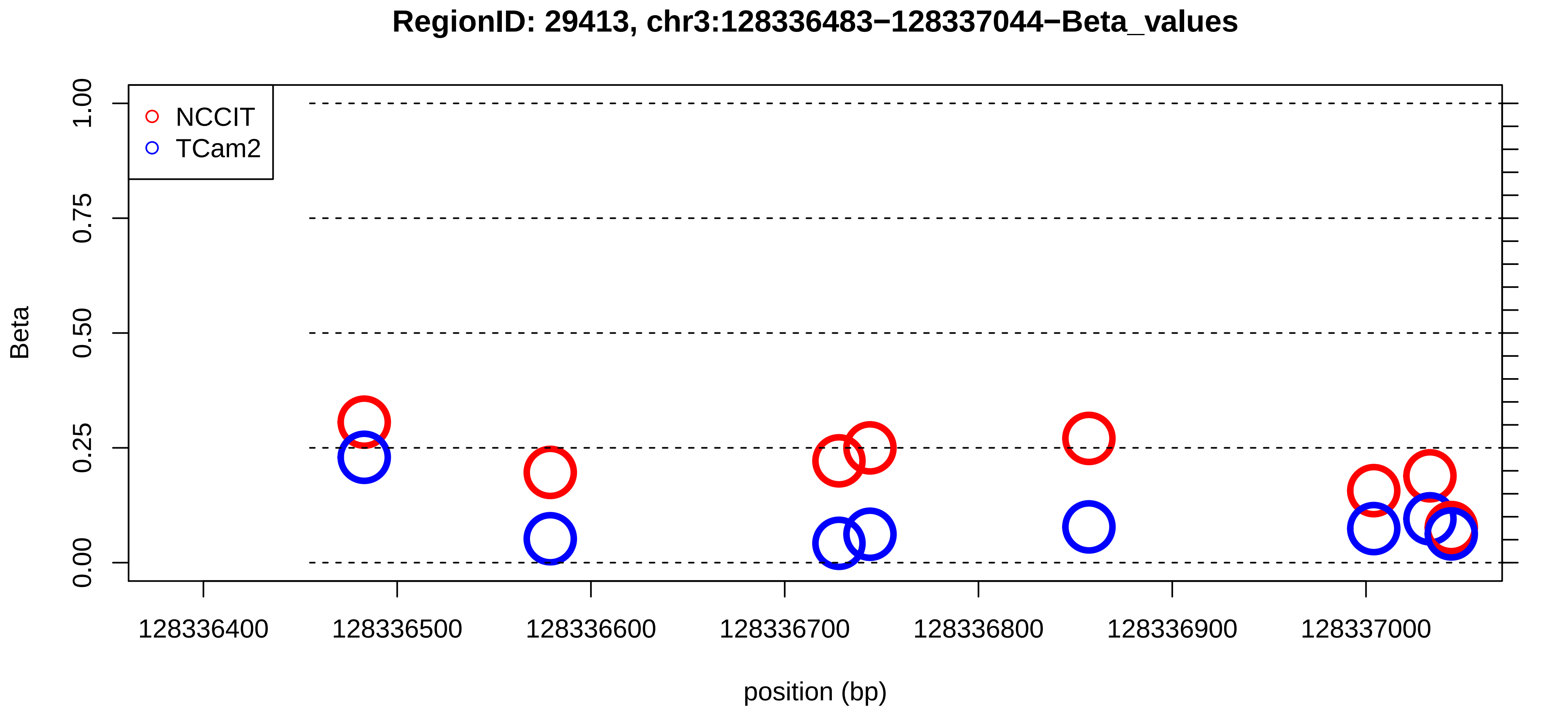

RegionID: 29413, chr3:128336483−128337044−Beta_values
NCCIT
TCam2
Beta
0.00
0.25
0.50
0.75
1.00
128336400
128336500
128336600
128336700
128336800
128336900
128337000
position (bp)
